# SIRT2‐knockdown rescues GARS‐induced Charcot‐Marie‐Tooth neuropathy

**DOI:** 10.1111/acel.13391

**Published:** 2021-05-30

**Authors:** Yingying Zhao, Liangguo Xie, Chao Shen, Qian Qi, Yicai Qin, Juan Xing, Dejian Zhou, Yun Qi, Zhiqiang Yan, Xinhua Lin, Rongyang Dai, Jinzhong Lin, Wei Yu

**Affiliations:** ^1^ State Key Laboratory of Genetic Engineering School of Life Sciences Zhongshan Hospital Fudan University Shanghai China; ^2^ School of Basic Medical Science Southwest Medical University Luzhou China

**Keywords:** *Drosophila* model, GARS, peripheral neuropathy, SIRT2

## Abstract

Charcot‐Marie‐Tooth disease is the most common inherited peripheral neuropathy. Dominant mutations in the glycyl‐tRNA synthetase (GARS) gene cause peripheral nerve degeneration and lead to CMT disease type 2D. The underlying mechanisms of mutations in GARS (GARS^CMT2D^) in disease pathogenesis are not fully understood. In this study, we report that wild‐type GARS binds the NAD^+^‐dependent deacetylase SIRT2 and inhibits its deacetylation activity, resulting in the acetylated α‐tubulin, the major substrate of SIRT2. The catalytic domain of GARS tightly interacts with SIRT2, which is the most CMT2D mutation localization. However, CMT2D mutations in GARS cannot inhibit SIRT2 deacetylation, which leads to a decrease of acetylated α‐tubulin. Genetic reduction of *SIRT2* in the *Drosophila* model rescues the GARS‐induced axonal CMT neuropathy and extends the life span. Our findings demonstrate the pathogenic role of SIRT2‐dependent α‐tubulin deacetylation in mutant GARS‐induced neuropathies and provide new perspectives for targeting SIRT2 as a potential therapy against hereditary axonopathies.

## Graphical abstract

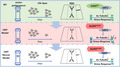

**Abbreviations**
CMT2D: Charcot‐Marie‐Tooth disease type 2D
GARS: glycyl‐tRNA synthetase
HDACs: histone deacetylases
SIRT2: sirtuin 2

## 1 INTRODUCTION

Charcot‐Marie‐Tooth disease is the most common inherited peripheral neuromuscular disorder, affecting 1 of every 2500 persons (Patzkó and Shy, 2011; Skre, 1974). A well‐organized microtubule network is required for peripheral axons to transport vesicles between the soma and the synapse effectively. The acetylation of α‐tubulin within the microtubules promotes the anchoring of the molecular motor kinesin and stimulates vesicular transport. Additionally, acetylated microtubules are far more stable and resistant to drug‐induced depolymerization than non‐acetylated microtubules (Matsuyama et al., 2002). Defects in axonal transport are often associated with neurodegeneration and with peripheral neuropathies in particular (Saxena & Caroni, 2011).

SIRT2 belongs to the class III HDACs and controls several proteins’ acetylation status, including α‐tubulin (North et al., 2003). Targeting the activity of SIRT2 is beneficial in Parkinson's disease (PD) and Huntington's disease (HD) but not in CMT (Donmez & Outeiro, 2013). Two studies have reported that targeting HDAC6, another deacetylase of α‐tubulin, may be of potential therapeutic benefit in the GARS‐induced CMT disease (Benoy et al., 2018; Mo et al., 2018). Although SIRT2 can deacetylate α‐tubulin in vitro (North et al., 2003), knockout of SIRT2 in mice does not alter the acetylation levels of α‐tubulin in vivo (Bobrowska et al., 2012; Taes et al., 2013), indicating that SIRT2 may deacetylate α‐tubulin under particular conditions. For example, SIRT2, not HDAC6, is responsible for the deacetylation of α‐tubulin during inflammasome activation in mice macrophages (Misawa et al., 2013). Here, we provide another evidence of SIRT2 knockdown rescuing the GARS‐induced CMT diseases.

Dominant mutations in the glycyl‐tRNA synthetase (GARS) gene cause peripheral nerve degeneration and lead to type 2D CMT disease. Notably, genetic studies in mice models of GARS^P234KY^ and GARS^C157R^ and a fly model of GARS^G240R^ revealed that these dominant mutations in GARS could cause CMT through toxic gain‐of‐function effects (Grice et al., 2015; Motley et al., 2011). Several studies have demonstrated that mutations in GARS (GARS^CMT2D^) that result in tRNA‐charging deficits play a role in disease pathogenesis, suggesting that partial loss of aminoacylation activities is involved (Antonellis et al., 2003, 2006; Griffin et al., 2014), but the underlying mechanisms are not fully understood.

This study reports that wild‐type GARS binds the NAD^+^‐dependent deacetylase SIRT2 and inhibits its deacetylation activity, resulting in maintaining hyperacetylated α‐tubulin, the primary substrate of SIRT2. Previous studies showed that the acetylation of α‐tubulin protects microtubules from mechanical breakage (Portran et al., 2017; Xu et al., 2017) and maintains axonal transportation (Godena et al., 2014). We performed the truncated IP assays to identify that the catalytic domain of GARS interacts with SIRT2, which is the most CMT2D mutation localization. These results imply that CMT2D mutations in GARS could alter its structural conformation, enabling GARS^CMT2D^ to lose binding with SIRT2 and reduce the inhibition of SIRT2 deacetylation, which leads to a decrease of acetylated α‐tubulin. The knockdown of *SIRT2* in a *Drosophila* model rescues GARS‐induced axonal CMT neuropathy and extends the life span. Our findings demonstrate the pathogenic role of SIRT2‐dependent α‐tubulin deacetylation in mutant GARS‐induced neuropathies and provide new possibilities for targeting SIRT2 as a potential therapy against hereditary axonopathies.

## 2 RESULTS

### 2.1 Wild‐type GARS tightly binds the SIRT2 to inhibit its activity, not GARS^CMT2D^

**FIGURE 1:**
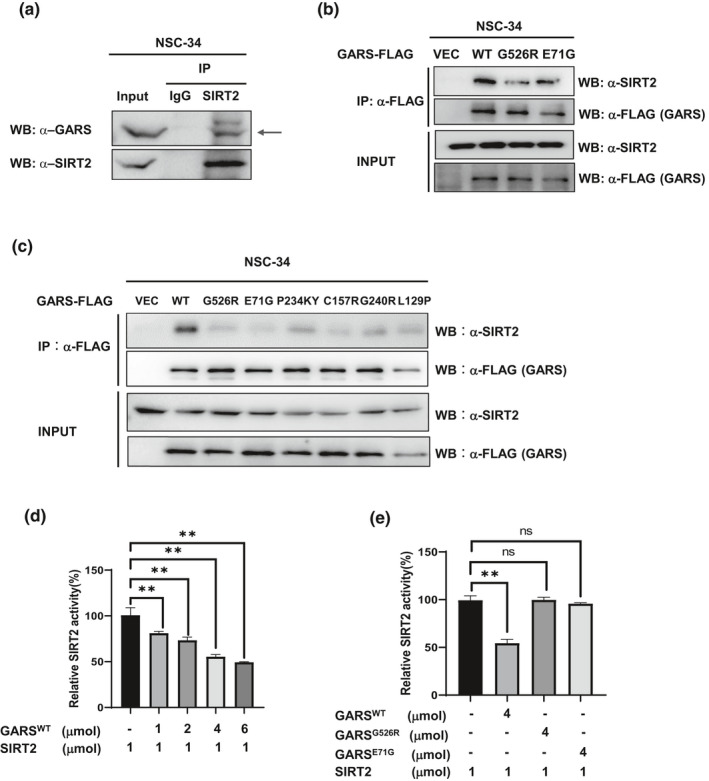
Wild‐type GlyRS tightly binds the SIRT2 to inhibit its activity, not GlyRS^CMT2D^. (a) Coimmunoprecipitation of endogenous sirt2 showing specifically interaction with endogenous GlyRS in NSC‐34 cells. (b) Representative immunoblotting of 3 independent experiments shows that SIRT2 interacts with wild‐type GlyRS, not GlyRS mutant in stably expressing FLAG‐tagged GlyRS NSC34 cells FLAG‐tagged GlyRS was stably expressed in NSC34 cells. Precipitated GlyRS‐FLAG (WT, G526R, E71G) was detected by anti‐FLAG antibody, and co‐IP endogenous SIRT2 was detected by anti‐SIRT2 as indicated. (c) Representative immunoblotting of 3 independent experiments shows that SIRT2 interacts with wild‐type GlyRS, not GlyRS mutants in NSC‐34 cells. The NSC‐34 cells were transfected with GlyRS‐FLAG (WT, G526R, E71G, P234KY, C157R, G240R, L129P). Coimmunoprecipitations were performed with anti‐FLAG M2 magnetic beads. The immunoblot analysis was performed with anti‐Sirt2 and anti‐FLAG. (d‐e) Effect of GlyRS(d) or GlyRS mutant (G526R, E71G) (e) on SIRT2 deacetylation activity. SIRT2 (1 µM) was incubated with purified GlyRS or GlyRS (G526R, E71G) (concentration measured as a monomer) at the indicated ratios. The deacetylase activities of recombinant human SIRT2 were measured by monitoring the fluorescence intensity (excitation at 360 nm and emission at 460 nm) using a substrate peptide with one end coupled to a fluorophore and the other end to a quencher. A reaction without NAD^+^ was performed as a negative control. Data are presented as mean ± SD, *n* = 3 biological replicates per group, from three independent experiments

Targeting SIRT2 has been shown to benefit other neurodegenerative diseases (Donmez & Outeiro, 2013). We want to test whether SIRT2, a major deacetylase of α‐tubulin, might also play a critical role in CMT diseases. We performed a coimmunoprecipitation assay to determine whether SIRT2 might interact with GARS or GARS^CMT2D^ mutants. First, we confirmed that endogenous SIRT2 could pulldown the endogenous GARS in NSC‐34 cells (Figure 1A), not SIRT1, the other Sirtuin family member in the cytoplasm (Figure S1A). We also generated stable expressing FLAG epitope‐tagged GARS (wild‐type and CMT2D mutants‐G526R, E71G) in NSC‐34 motor neuron cells. We found that GARS^WT^ could pull down more endogenous SIRT2 than GARS^G526R^ and GARS^E71G^ (Figure 1B). To explore whether most of the CMT2D mutants in GARS might lose the interaction with SIRT2, we performed the co‐IP assays in NSC‐34 and found only GARS^WT^ significantly binds with SIRT2, and all the GARS^CMT2D^ reduce the binding ability to SIRT2 (Figure 1C). Moreover, in HEK293T cells, SIRT2 interacted tightly with GARS^WT^ but not with the GARS^CMT2D^ mutants (G526R, E71G) (Figure S1B,C). These results indicated that it might be a general phenomenon that GARS^WT^, not GARS^CMT2D^, could tightly interact with SIRT2. To determine whether the interaction of GARS^WT^ with SIRT2 would directly affect the deacetylase activity of SIRT2, we purified SIRT2 and GARS from *E*. *coli* to perform an in vitro deacetylase assay. We tested the effect of different GARS concentrations on SIRT2 activity and found that GARS significantly decreased SIRT2 activity in a dose‐dependent manner (Figure 1D), while the same concentrations of GARS^CMT2D^ barely inhibited SIRT2 deacetylase activity (Figure 1E). The data demonstrate that the GARS interaction directly inhibits SIRT2 deacetylase activity in a dose‐dependent manner.

### 2.2 Wild‐type GARS binds the SIRT2 to maintain the acetylated a‐tubulin

**FIGURE 2:**
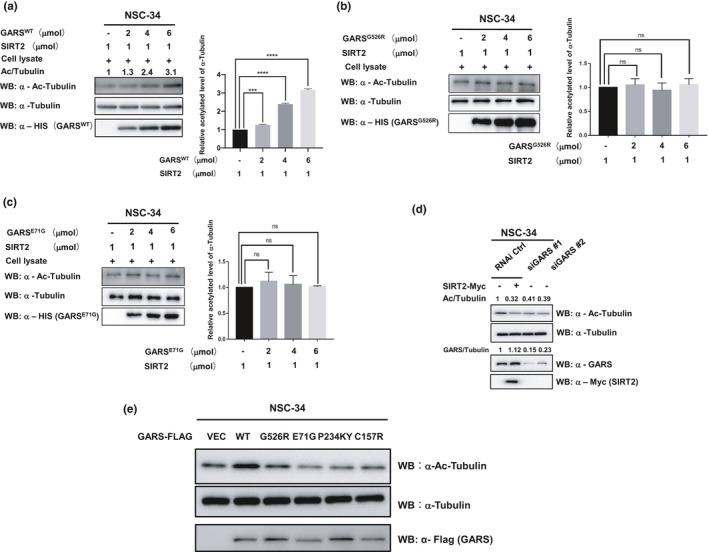
Wild‐type GlyRS binds the SIRT2 to maintain the acetylated a‐tubulin. (a–c) The recombinant SIRT2(1µmol) and recombinant GlyRSWT (a), GlyRSG526R (b), or GlyRSE71G (c) were incubated with NSC‐34 lysate at the indicated ratios(concentration measured as a monomer). The reaction products were detected by Western blotting for acetylated tubulin, a‐tubulin, and His‐tag. Data are presented as mean ± SD, *n* = 3 biological replicates per group, from three independent experiments. (d) Representative immunoblotting of 3 independent experiments shows that both knockdowns of GlyRS and overexpression of SIRT2 result in decreased tubulin acetylation. HEK293 cells were transfected with GlyRS siRNA or control siRNA 24 h later, and SIRT2‐Myc was overexpressed in cells transfected with control siRNA. After an additional 48 h, cells were harvested and acetylation of tubulin was detected by Western blot. (e) Western blot analysis detecting the level of α‐tubulin acetylation in NSC‐34 cells transfected with wild‐type GlyRS and GlyRS mutants

Since SIRT2 is responsible for deacetylating α‐tubulin under particular conditions (Misawa et al., 2013; Nagai et al., 2013), we confirmed that GARS, SIRT2, and α‐tubulin are major localizing in the cytoplasm of NSC‐34 cells (Figure S1D). To demonstrate that the inhibition of SIRT2 affects the acetylation of α‐tubulin in vitro, we performed an in vitro analysis by incubating recombinant SIRT2 and NSC‐34 or HEK293T cellular lysates with increased concentrations of GARS^WT^, GARS^G526R,^ and GARS^E71G^. We observed a significantly increased level of α‐tubulin acetylation when cellular lysates and SIRT2 were incubated with wild‐type GARS in a dose‐dependent manner (Figure 2A and Figure S1E), but not in the GARS^G526R^ and GARS^E71G^ (Figure 2B,C, Figure S1F). We also knockdown GARS and detected the level of α‐tubulin acetylation in NSC‐34 cells expressing two different GARS siRNA. The acetylation levels of α‐tubulin in the two different GARS knockdown cells were significantly decreased, which were similar to those resulting from the overexpression of SIRT2 in cells (Figure 2D). To understand whether the tight interaction of GARS might affect the deacetylation of SIRT2 on α‐tubulin in cells, we overexpressed GARS^WT^ and GARS^CMT2D^ in NSC‐34 cells and then detected the level of α‐tubulin acetylation in cells. We found GARS^WT^ increases the level of α‐tubulin acetylation but GARS^CMT2D^ significantly decreases the acetylated α‐tubulin (Figure 2E). These data indicate that the interaction of wild‐type GARS and SIRT2 inhibits the deacetylase activity of SIRT2 and leads to the acetylated α‐tubulin, which might protect microtubules from mechanical breakage.

### 2.3 The catalytic domain of GARS tightly binds the SIRT2

**FIGURE 3:**
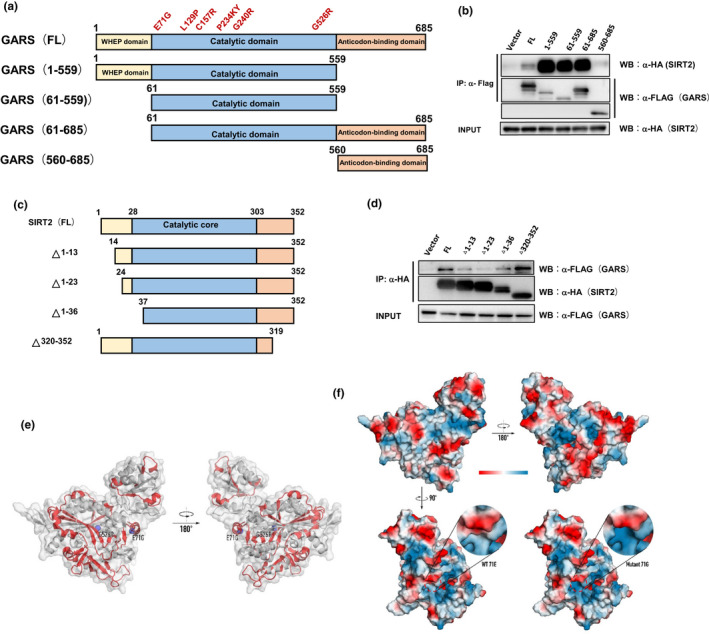
The catalytic domain of GlyRS tightly binds the SIRT2. (a, b) Mapping study to identify the SIRT2 binding sites on GlyRS. FLAG‐tagged full‐length GlyRS or its truncated fragments was co‐transfected with HA‐tagged SIRT2 into HEK 293 cells. GlyRS proteins were immunoprecipitated with anti‐FLAG antibody, and the GlyRS ‐bound SIRT2 proteins were detected by Western blot using anti‐HA antibody. (c‐d) Mapping study to identify the GlyRS binding sites on SIRT2. HA‐tagged full‐length SIRT2 or its truncated fragments was co‐transfected with FLAG‐tagged GlyRS into HEK 293 cells. SIRT2 proteins were immunoprecipitated with anti‐HA antibody, and the SIRT2‐bound GlyRS proteins were detected by Western blot using anti‐FLAG antibody. (e) Structure of human GlyRS (PDB: 2ZT5) with the opened surface (red) caused by G526R and E71G mutations. (f) Electrostatic potential maps of WT GlyRS or GlyRS with E71G mutation. All images generated with PyMol

To explore the mechanism of wild‐type GARS binds with SIRT2, we performed the truncated immunoprecipitation assay to map GARS and SIRT's binding domain. We found that the catalytic domain of GARS tightly interacts with SIRT2 (Figure 3A,B). Considering the most CMT2D mutants localize in the catalytic domain of GARS, these data could explain GARS^CMT2D^ disrupting the binding with SIRT2. Reversely, we found that the N‐terminal end of SIRT2 is critical for interaction with GARS. The C‐terminal end of SIRT2 could prevent the GARS from interacting with the catalytic core of SIRT2. These require us to reach the crystal structure to know how wild‐type GARS binds and inhibits SIRT2 activity more precisely. We inspected the 3D structure of the human GARS protein (PDB: 2ZT5) and found both CMT2D mutations caused a conformational opening surface in GARS (Figure 3E). Compared with E71 GARS having negatively charged regions, the E71G mutant represents neutral charged regions (Figure 3F). Together, these data made us hypothesize that structural alteration induced by CMT2D mutations in GARS might disrupt the proper binding with SIRT2.

### 2.4 SIRT2 knockdown rescues CMT phenotype in GlyRS^G526R^ flies

**FIGURE 4:**
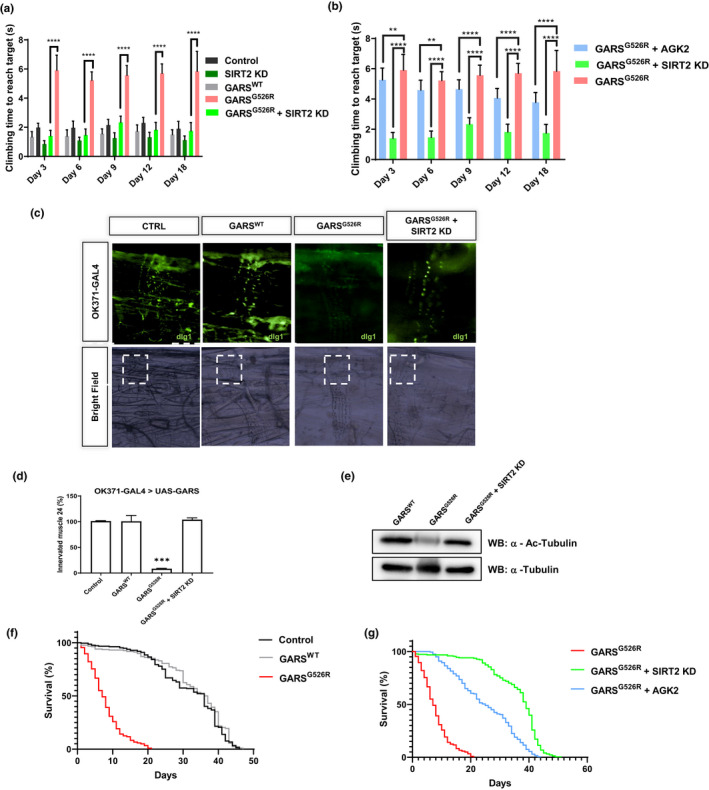
SIRT2 knockdown rescues CMT phenotype and life span in GlyRSG526R flies. (a) Bar graph displaying average climbing time to reach the target in a negative geotaxis assay of female flies in motor neurons (OK371‐GAL4). WT (gray), SIRT2 RNAi in GlyRSG526R (green), and GlyRSG526R (pink). *N* > 100. Error bars represent SEM. ****p < 0.0001. (b) 100 µM AGK2 was fed from 12‐h flies to 3‐ to 18‐day‐old flies; then, motor performance was detected and the bar graph was displayed average climbing time. AGK2‐fed GlyRSG526R (blue), SIRT2 RNAi in GlyRSG526R (green), and GlyRSG526R (pink). 4% DMSO was fed in all lines. *N* > 100. Error bars represent SEM. ****p < 0.0001. (c) SIRT2 knockdown restores NMJ in GlyRSG526R mutants. NMJs of third instar larvae expressing GlyRS in motor neurons (OK371‐GAL4) were visualized by staining for the postsynaptic marker disks large 1 (dlg1). Results indicate the NMJ on muscle 4, which is missing in GlyRSG526R flies and is rescued in SIRT2 knockdown GlyRSG526R flies. Scale bar, 50 mm. (d) Quantification of the percentage of animals with muscle 24 innervated; c2‐Test; ***p < 1 × 10^−6^; *N* = 25. (e) Representative immunoblotting of 3 independent experiments shows that acetylated α‐tubulin in the lysate of different fly lines. (f) UAS‐SIRT2‐RNAi lines (BL31613) were bought from Tsinghua Fly Center and crossed with tub‐Gal4 as the control (UAS‐SIRT2 RNAi/+) for the longevity assay. Kaplan–Meier survival curves displaying the lifespan of male flies from the adult stage onwards. GlyRSG526R flies have a shorter median life span than GlyRSWT flies and Tubts control flies (*p* < 0.0001, log‐rank test). *N* > 200 (g) SIRT2 knockdown or inhibitor extends the median life span of GlyRSG526R (*p* < 0.0001, log‐rank test). *N* > 200

A previous study showed that GARS with a CMT‐associated mutation‐induced motor performance deficits in a *Drosophila* model (Niehues et al., 2015). We were gifted the GARS^WT^, GARS^G526R,^ and GARS^E71G^ of fly models from Dr. Storkebaum (Niehues et al., 2015) and confirmed that the GARS^G526R^ fly model showed worse motor performance deficits than the GARS^E71G^ fly model and GARS^WT^ (Figure S2A), so we chose the GARS^G526R^ to perform the rescue experiments. To determine how SIRT2 affects a GARS mutant in vivo, we assessed the motor behavior resulting from *sirt2*‐knockdown in GARS^G526R^ flies. We generated SIRT2 knockdown in GARS^G526R^ flies (Figure S2B) and confirmed the knockdown efficiency of SIRT2 by real‐time PCR and Western blot (Figure S2C,D). Compared to GARS^G526R^ flies, GARS^G526R^ flies with *sirt2*‐knockdown showed significantly restored climbing ability and similar to WT flies (Figure 4A and Supplementary Figure S3A, Video S1). To provide further evidence that the inhibition of SIRT2 might rescue the phenotype of CMT‐mutant GARS in the *Drosophila* model, we fed GARS^G526R^ flies with AGK2, which is a specific inhibitor of SIRT2 (Outeiro et al., 2007). We then examined the climbing ability of these treated GARS^G526R^ flies. We found that 100 µM AGK2‐fed GARS^G526R^ flies showed significantly shorter climbing times than DMSO‐fed GARS^G526R^ flies (Figure 4B and Supplementary Figure S3B, Video S2). Interestingly, the motor performance of GARS^G526R^ flies was gradually rescued by AGK2 treatment in GARS^G526R^ flies in a time‐dependent manner (Figure 4B). Since mutant GARS flies and mice also showed neuronal morphological defects(Niehues et al., 2015; Spaulding et al., 2016), we performed a staining analysis of neuromuscular junctions (NMJs) to assess the development status of NMJs in *sirt2*‐knockdown GARS^G526R^ flies. Consistent with a previous report that GARS^G526R^ flies showed a dramatic decrease in NMJs, we found that *sirt2*‐knockdown in GARS^G526R^ flies significantly increased NMJs’ numbers in third instar larvae to levels similar to those in WT flies (Figure 4C,D). Moreover, we observed the acetylated tubulin was significantly rescued in *sirt2*‐knockdown in GARS^G526R^ flies comparing with the GARS^G526R^ flies (Figure 4E). Overall, these results strongly indicate that the inhibition of SIRT2 could rescue the motor performance deficits in GARS‐mutant CMT flies in a *Drosophila* model.

A previous study showed that CMT‐mutant GARS flies have shortened lifespans (Niehues et al., 2015). To investigate whether SIRT2 knockdown or inhibition by AGK2 could affect CMT‐mutant GARS flies’ lifespan, we conducted a longevity analysis of *sirt2*‐knockdown GARS^G526R^ flies using a ubiquitously expressed driver (Tub > SIRT2^RNAi^) or 100 µM AGK2‐fed GARS^G526R^ flies. We first generated ubiquitous GARS transgene expression flies using the GAL80 target system. Consistent with a previous report that GARS^WT^ files did not show changes in their lifespan, GARSG526R flies showed significantly reduced lifespans than GARS^WT^ files (Figure 4F). Strikingly, the knockdown of *sirt2* expression or AGK2 significantly extended the median life span of GARS^G526R^ flies (Figure 4G). These data indicate that the loss of SIRT2 function in CMT‐mutant GARS flies resulted in a longer lifespan and strongly indicated the involvement of SIRT2 in CMT phenotype regulation.

## 3 DISCUSSION

**FIGURE 5:**
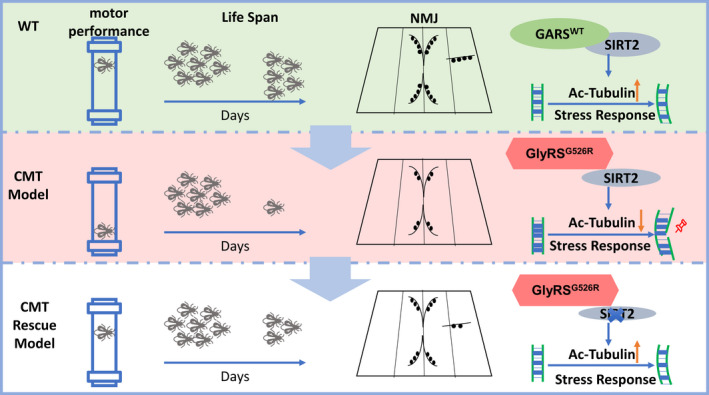
Schematics of targeting SIRT2 as a critical node between acetylated tubulin and CMT neuropathy

Targeting the expression or deacetylation activity of SIRT2 has been shown to benefit in several neurodegenerative diseases such as Parkinson's disease, Huntington's disease, and amyotrophic lateral sclerosis (ALS), but not shown benefit in CMT (Donmez & Outeiro, 2013). Mutations in five aminoacyl tRNA synthetases, including GARS, have been identified that cause CMT or related peripheral neuropathies (Storkebaum, 2016). In this study, we showed that WT GARS but not GARS^CMT2D^ binds to SIRT2 and prevents its deacetylation activity, which leads to the acetylated α‐tubulin. Our data revealed that CMT2D mutations in GARS could not inhibit the deacetylation activity of SIRT2, resulting in hypoacetylated α‐tubulin. Furthermore, the genetic reduction of *SIRT2* expression and a specific inhibitor of SIRT2 in a *Drosophila* model rescued the GARS‐induced axonal CMT neuropathy and extended the life span (Figure 5). Our study outlines a method for targeting SIRT2 to reduce the GARS‐induced CMT neuropathy.

Acetylated αtubulin has been shown to play multiple cellular functions, including the regulation of cell motility and polarity and participation in intracellular transport, ciliary assembly, and immune and viral responses (Li & Yang, 2015). Interestingly, the Maxence Nachury group recently reported that acetylated α‐tubulin protects microtubules from mechanical breakage (Portran et al., 2017; Xu et al., 2017), which may be linked to the neuropathy of CMT disease, which usually occurs in the first two decades of life because this stage in life, the hypoacetylated microtubules in peripheral motor neurons are not able to endure the mechanical stress and accumulated the lattice damage, resulting in the degeneration of peripheral motor and sensory axons. Hence, we demonstrated that SIRT2 knockdown could ameliorate the phenotype associated with CMT2D pathology by rescuing neuromuscular junctions in flies. This is an interesting outcome since defects in neuromuscular junction maturation precede the impaired connectivity of lower motor neurons in CMT2D mice (Sleigh et al., 2014; Spaulding et al., 2016).

SIRT2 localized in the cytoplasm and was identified as the deacetylase of lysine 40 in α‐tubulin in vitro (North et al., 2003). Compared with HDAC6, SIRT2 may function as a deacetylase of α‐tubulin in particular conditions, such as in the mitotic spindle (Nagai et al., 2013) or during the inflammasome activation in macrophages (Misawa et al., 2013). The inhibition of SIRT2 expression or deacetylase activity was shown to rescue a‐synuclein toxicity in a model of Parkinson's disease (Outeiro et al., 2007). Additionally, the pharmacological inhibition of SIRT2 could impair sterol biosynthesis to provide neuroprotection in models of Huntington's disease (Luthi‐Carter et al., 2010). CMT is one of the most common inherited neuropathy, usually occurs in the first two decades of life and shows progressive weakness and atrophy in hands and feet (Storkebaum, 2016). We found the wild‐type GARS could interact with SIRT2 and inhibit its deacetylase to maintain hyperacetylated α‐tubulin in HEK293T and NSC‐34 cells, implying this might be a general phenomenon in these cells. Since SIRT2 is most abundant in skeletal muscle and the aging central nervous system (Maxwell et al., 2011), we hypothesis these GARS mutants lose the binding with SIRT2 and result in a significant decrease in the acetylated tubulin in muscle and CNS, which might contribute the neuropathy like CMT. Our study provides another promising strategy to treat the peripheral neuropathy resulting from CMT by inhibiting SIRT2, suggesting that the targeting of SIRT2 might have a general beneficial impact on neurodegenerative diseases.

Two genetics experiments in flies and mice have currently demonstrated that dominant mutations in GARS cause CMT through toxic gain‐of‐function effects (Motley et al., 2011; Niehues et al., 2015). Several binding partners of CMT mutants have been discovered, including Nrp1 (He et al., 2015), Trk (Sleigh et al., 2017), and HDAC6 (Benoy et al., 2018; Mo et al., 2018). Recently, Mo et al. (2018) have shown that mutant GARS of P234KY could bind and activate HDAC6 deacetylation of α‐tubulin. However, we presented that SIRT2, the acetylated tubulin deacetylase, can bind tightly with wild‐type GARS compared with CMT mutants. We did not rule out the possibility that CMT mutants might gain the functions to increase the deacetylation activity of SIRT2, which leads to a decrease in the acetylated tubulin. Interestingly, HDAC6 can form a complex with SIRT2 (North et al., 2003), which made us hypothesize that CMT mutants might bind with HDAC6 to affect the deacetylation activity of SIRT2. This needs to be addressed in future experiments.

## 4 EXPERIMENTAL PROCEDURES

### 4.1 Antibodies

The anti‐SIRT2 antibody was purchased from Abcam (ab51023) and Proteintech (19655‐1‐AP). Anti‐GlyRS antibody was purchased from Proteintech (15831‐1‐AP). Antibodies against α‐tubulin and acetylated tubulin at Lys40 (TubulinK40Ac) were purchased from Invitrogen (62204) and Sigma (T6793), respectively. Antibodies to FLAG (Sigma, SAB4301135), HA (Abcam, ab9110), Myc (Abcam, ab9106), and His (abmart 293670) were commercially obtained.

### 4.2 Plasmid construction

For overexpression in mammalian cells, human GARS genes were cloned into the pcDNA‐3.1‐FLAG vector and pcDNA3.1‐MYC vector, and human SIRT2 genes into the pRK7‐HA vector and pcDNA‐3.1‐FLAG vector. Point mutations for GlyRS were generated by site‐directed mutagenesis (Toyobo KOD Mut Kit). Human GlyRS and SIRT2 genes were cloned into the pET‐28a(+) to express with a C‐terminal his‐tag in Escherichia coli.

### 4.3 Cell culture and transfection

NSC‐34 motor neuron cells and HEK293T cells were cultured in DMEM/high glucose medium (HyClone) supplemented with 10% fetal bovine serum (BI), 100 units/ml penicillin, and 100 mg/ml streptomycin (Sangon Biotech). All cells were cultivated in a humidified incubator with 5% CO2 at 37°C. According to the manufacturer's protocol, cell transfection except for NSC‐34 cells and siRNA was carried out by polyethylenimine (PEI). Cell transfection for NSC‐34 cells and siRNA was carried out by Lipofectamine 2000 according to the manufacturer's protocol. All transfections were done when cells reach 50%–70% confluence.

The sequences of siRNAs were as follows:

siRNA_GARS1, 5′‐ CTTGAGACCAGAAACTGCA‐3′; siRNA_GARS2, 5′‐ GTAGCTGAGAAACCTCTGA‐3′.

### 4.4 Coimmunoprecipitation assays

SIRT2 and GlyRS were co‐transfected into HEK293T cells, and cell lysates were immunoprecipitated with FLAG beads (Sigma Aldrich) overnight at 4°C and then boiled with SDS loading buffer and subjected to Western blotting. SIRT2‐FLAG and MYC‐tagged GlyRS or mutant GlyRS were detected as indicated.

### 4.5 Cell lysis and Western Blot analysis

For cell‐based experiments, cells were washed three times in PBS and resuspended with lysis buffer containing 50 mM Tris, pH 7.5, 150 mM NaCl, 1% Nonidet P‐40, 1 mg/ml aprotinin, 1 mg/ml leupeptin, 1 mg/ml pepstatin, 1 mM Na3VO4, and 1 mM phenylmethylsulfonylfluoride (PMSF), and 25 mM NAM and trichostatin A (TSA) for 20 min and centrifuged for 20 min at 13,800 *g*; the insoluble fraction was discarded. The lysates were fractionated by SDS/PAGE and transferred to nitrocellulose filter (NC) membranes. The membranes were blocked for 1 h with Tris‐buffered saline with Tween 20 (TBST) containing 5% (mass/vol) nonfat dry milk. After incubation with primary antibodies (anti‐acetylated tubulin (K40) (Proteintech), anti‐α‐tubulin (Invitrogen), anti‐SIRT2(Abcam) or anti‐His (Abmart), each diluted 1:1000), the membranes were washed and incubated with HRP‐conjugated anti‐mouse (Cell Signaling Technology) or anti‐rabbit secondary antibodies (Cell Signaling Technology), followed by detection using ECL Western blotting substrate (Bio‐Rad).

### 4.6 SIRT2 deacetylase activity assay

The reaction buffer contains 50 mM Tris‐HCl (pH 9.0), 4 mM MgCl2, 0.5 mM DTT, 1 µM MAL(Boc‐Lys(AC)‐AMC), 1 mM NAD+, 1 µM recombinant SIRT2, and recombinant GlyRS or GlyRS mutant at different ratio indicated in Figures 1b. The reactions were performed at room temperature. Add the stop solution (0.05 g/ml trypsin) of the same volume as the reaction system to stop the reaction, and measure fluorescence intensity as indicated.

### 4.7 Purification of Recombinant SIRT2, GlyRS^WT,^ and GlyRS^CMT2D^ mutants

All genes were PCR‐amplified and cloned into the pET28a vector to produce His6‐tag fused recombinant proteins. Point mutations were introduced by the site‐directed mutagenesis approach. All recombinant proteins used in this study were expressed in *E. coli* BL21 (DE3) induced by 0.5 mM IPTG at 16°C for 20 h and collected by sedimentation. To purify wild‐type GlyRS and the G526R GlyRS mutants, the *E. coli* cells were resuspended in binding buffer (20 mM Tris‐Cl pH 8.0, 500 mM NaCl, and 25 mM imidazole), lysed with a high‐pressure homogenizer, and sedimented at 13,800 *g* for 1 h to pellet the debris. The supernatant lysates were purified by HisTrapTM FF. Then, the proteins were further purified on an AKTA purifier (GE Healthcare) and eluted with elution buffer (20 mM Tris‐Cl pH 8.0, 500 mM NaCl, and 500 mM imidazole). All the purified proteins were concentrated by centrifugal filtrations and then stored in aliquots at −80°C.

### 4.8 In Vitro α‐tubulin deacetylation assay

For SIRT2 experiments (Figure 2), the corresponding ratio (1/2, 1/4, 1/6) of recombinant SIRT2(1μmol) and GlyRSWT or GlyRSCMT2D mutants was resuspended in 100 μl of SIRT deacetylase buffer (50 mM Tris‐HCI, 4 mM MgCl2, 0.2 mM DTT, [pH 9.0]) added with 100 μg of total cellular lysate from untransfected NSC34 or HEK293T cells. Reactions were preincubated for 15 min and incubated for 1 hr at 37°C after the addition of 1 mM NAD^+^. A reaction is added with 5 mM nicotinamide as a negative control. Reactions were stopped by adding 34 μl of 5× SDS‐PAGE loading buffer. 8 μl of each reaction was separated on 12% SDS‐PAGE gels and Western blotted as described above.

### 4.9 Cytoplasmic and nuclear fractionation

One 10‐cm‐diameter plate of NSC‐34 cells was lysed in1 mL of lysis buffer containing 10 mM Hepes, pH 7.9, 50 mM NaCl, 0.5 M sucrose, 0.1 mM EDTA, 0.1% Triton X100, and freshly added multiple protease inhibitors (1% Nonidet P‐40, 1 mg/ml aprotinin, 1 mg/ml leupeptin, 1 mg/ml pepstatin, 1 mM Na3VO4, and 1 mM phenylmethylsulfonylfluoride (PMSF), 25 mM NAM and trichostatin A) at 4°C for 20 min and then centrifuged at 500 g for 15 min to pellet the nucleus. The pellet was washed three times with washing buffer containing 10 mM Hepes, pH 7.9, 10 mM KCl, 0.1 mM EDTA, 0.1 mM EGTA, and freshly added above protease inhibitors. The supernatant was subjected to 17,000 g centrifugation for another 10 min to remove any nuclear contamination and transferred to a new tube. The pellet and supernatant were boiled separately in SDS sample buffer.

### 4.10 Drosophila genetics

The OK371‐GAL4 driver line was kindly provided by Pro. Junhai Han from Southeast University, and other GAL4 driver lines were obtained from Prof. Xinhua Lin from Fudan University. The dGARS transgenetic lines of fly strains described in this paper were kindly gifted from Prof. Erik Storkebaum in Max Planck Institute for Molecular Biomedicine, which are transgenic strains overexpressing human wild‐type or pathogenic GARS mutations. UAS‐SIRT2‐RNAi lines (BL31613) were bought from Tsinghua Fly Center and crossed with tub‐Gal4 as the control (UAS‐SIRT2 RNAi/+) for the longevity assay.

### 4.11 Lifespan analysis

For the determination of adult offspring frequencies, the number of adult flies eclosing was counted for each genotype. For assaying longevity, the tub‐Gal4 driver was combined with a ubiquitously expressed temperature‐sensitive Gal80 inhibitor (tub‐Gal80ts). Fly crosses were cultured at 19°C, and adult progeny carrying the tubGal4, tub‐Gal80ts, and UAS‐GARS transgenes were shifted to 30°C to induce transgene expression. Males were collected within 24 h of eclosion and grouped into batches of 20 flies per food vial. The number of dead flies was counted every day, and flies were transferred to fresh food vials every 2 days. At least 200 flies per genotype were used.

### 4.12 Analysis of neuromuscular junction

For observation of neuromuscular junction, it was dissected from third instar larvae that selectively express target genes in motor neurons (OK371‐GAL4). Larval filets were prepared and stained with primary antibodies against dlg1 (DSHB. 1/200).

### 4.13 Drosophila behavior analysis

Flies for motor performance assays were kept at 25°C with a 12‐h light/dark cycle. Female flies were collected within 24 h after eclosion and divided into groups of 20 individuals. Motor performance of 3‐ to 18‐day‐old flies was evaluated. On the day of the assay, flies were transferred in test tubes without anesthesia and assayed within 15 min under standardized daylight conditions. Three test tubes were loaded into a selfmade device, which was released from a certain height. The device fell onto the table, shaking the flies to the bottom of the test tubes and inducing a negative geotaxis climbing response. The whole procedure was videotaped with a camera and repeated five more times. The average climbing time to reach 6 cm was determined and compared between genotypes. At least 100 flies per genotype were used.

### 4.14 Isolation of total RNA from fly larvae

Total RNA was isolated from third instar larvae under acidic conditions using TRIzol. Approximately 10 frozen larvae were covered with 5 ml of liquid nitrogen. All subsequent steps were carried out on the ice or at 4°C. And 1 ml of TRIzol was added to the homogenate, and the mixture was vortexed for 30 s followed by chilling on ice for 5 min. An amount of 0.3 ml of chloroform was added and mixing the samples repeatedly. After centrifugation at 12,000 g for 10 min, the aqueous phase was transferred to a new tube. RNA was precipitated by 0.5 ml isopropanol by chilling on ice for 10 min. The resulting RNA pellet was washed twice with 75% ethanol. RNA was resuspended in DEPC and stored at 80°C.

### 4.15 Statistical analysis

We used GraphPad Prism 6 software for statistical calculations. An unpaired *t* test was used to analyze offspring frequency and behavior data. All data are reported as the mean SEM.

### 4.16 Fly information

**Table:** 

Identifiers	Designation	Source or reference
	Tub‐Gal4, Tub‐Gal80ts	Li et al (2013)
	OK371‐Gal4	Han et al. (2015)
EST1397	yw or w; UAS_hGARS_wt_vk37.9.2/CyO	Niehues et al., (2015)
EST1412	yw or w; UAS_hGARS_G526R_vk37.5.1/CyO	Niehues et al., (2015)
THU1950 (BL31613)	UAS‐SIRT2 RNAi	Tsinghua Fly Center

## CONFLICT OF INTEREST

The authors declare no competing interests.

## AUTHOR CONTRIBUTIONS

W. Yu designed the study and analyzed data. Y. Zhao, L. Xie, C. Shen, Q. Qi, Y. Qin, J. Xing, and R. Dai performed biochemistry experiments and analyzed data; D. Zhou and J. Lin worked the structural analysis; C. Shen and Y. Zhao worked the fly experiments under the assistance of Y. Qi, Z. Yan, and X. Lin. Y. Zhao, L. Xie, C. Shen and W. Yu wrote the paper.

## Supporting information

Fig S1‐S3

Video S1

Video S2

## Data Availability

The data that support the findings of this study are available from the corresponding author upon reasonable request.
