# Supplementary material for: SIRT2‐knockdown rescues GARS‐induced Charcot‐Marie‐Tooth neuropathy
Source: Aging Cell. 2021 May 30;20(6):e13391. doi: 10.1111/acel.13391 (PMC8208790; doi:10.1111/acel.13391)
Supplement: Supplementary file 1 — Fig S1‐S3 [file ACEL-20-e13391-s002.pdf]

Supplemental Figure 1

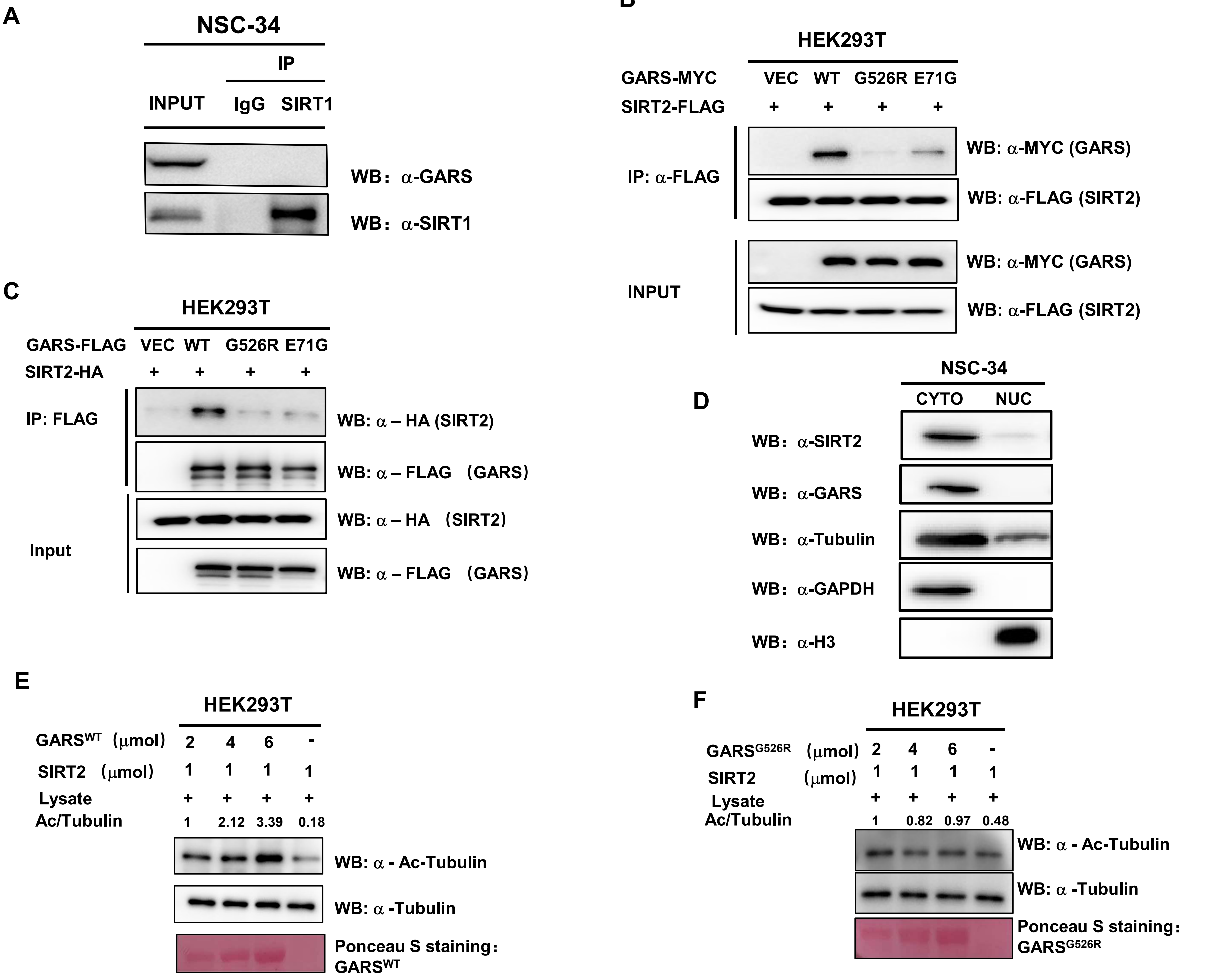

Supplemental Figure 2

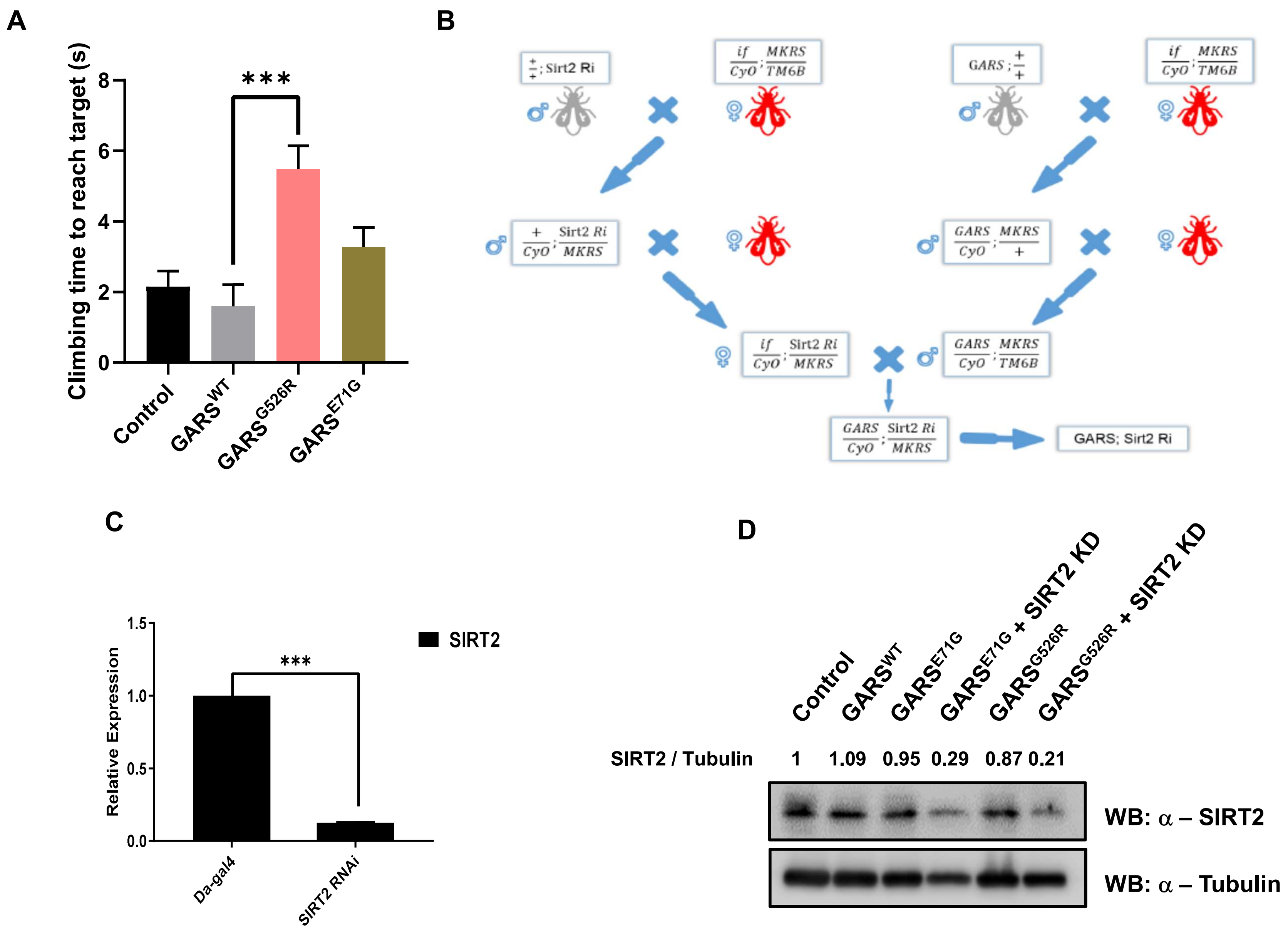

Supplemental Figure 3

A

L: WT

M:GARS^{G526R}+SIRT2 KD

R: GARS^{G526R}

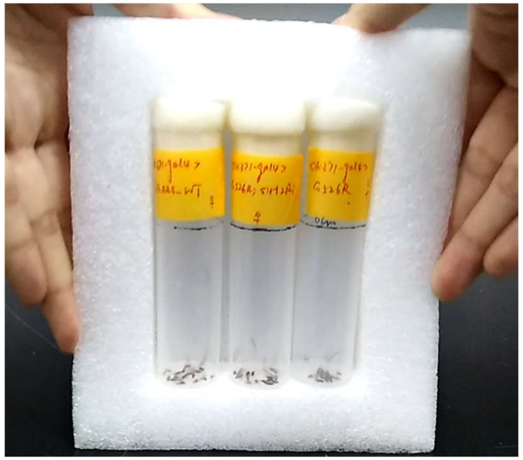

Day 10

B

L: GARS^{G526R}+AGK2

M: GARS^{G526R} +SIRT2 KD

R: GARS^{G526R}

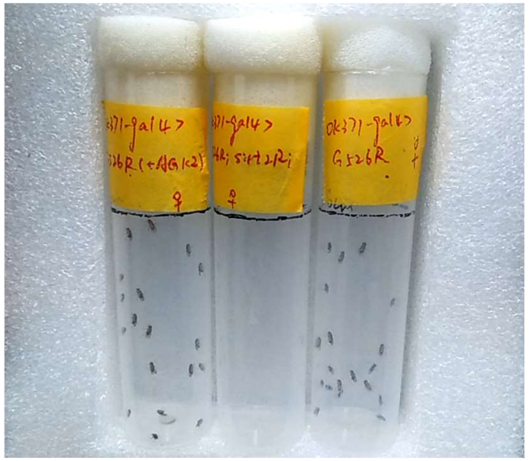

Day 10
